# Identification of B-Cell Epitopes with Potential to Serologicaly Discrimnate Dengue from Zika Infections

**DOI:** 10.3390/v11111079

**Published:** 2019-11-19

**Authors:** Alice F. Versiani, Raissa Prado Rocha, Tiago A. O. Mendes, Glauco C. Pereira, Jordana Graziella A. Coelho dos Reis, Daniella C. Bartholomeu, Flávio G. da Fonseca

**Affiliations:** 1 Laboratório de Virologia Básica e Aplicada, Departamento de Microbiologia, Universidade Federal de Minas Gerais, Belo Horizonte 31270-901, Brazil; afversiani@gmail.com (A.F.V.); raissa.biotec@gmail.com (R.P.R.); reisjordana@gmail.com (J.G.A.C.d.R.); 2 Laboratório de Pesquisa em Virologia, Departamento de Doenças Infecciosas e Parasitárias, Faculdade de Medicina de São José do Rio Preto, São José do Rio Preto 15090-000, Brazil; 3 Laboratório de Imunologia e Genômica de Parasitos, Departamento de Parasitologia, Universidade Federal de Minas Gerais, Belo Horizonte 31270-901, Brazil; tiagomgmendes@yahoo.com.br (T.A.O.M.); daniellaufmg@gmail.com (D.C.B.); 4 Fundação Ezequiel Dias, FUNED, Belo Horizonte 30510-010, Brazil; glaiconep@gmail.com

**Keywords:** dengue diagnose, Zika, peptides, epitopes, pepELISA

## Abstract

Dengue is currently one of the most important arbovirus infections worldwide. Early diagnosis is important for disease outcome, particularly for those afflicted with the severe forms of infection. The goal of this work was to identify conserved and polymorphic linear B-cell Dengue virus (DENV) epitopes that could be used for diagnostic purposes. To this end, we aligned the predicted viral proteome of the four DENV serotype and performed *in silico* B-cell epitope mapping. We developed a script in Perl integrating alignment and prediction information to identify potential serotype-specific epitopes. We excluded epitopes that were similarly present in the yellow fever and zika viruses’ proteomes. A total of 15 polymorphic and nine conserved peptides among DENV serotypes were selected. Peptides were spotted on cellulose membranes and tested against sera from rabbits that were monoinfected with each DENV serotype. Although serotype-specific peptides failed to recognize any sera, three conserved peptides were recognized by all anti-dengue sera and were included on an ELISA test employing a well-characterized human sera bank. Of the three peptides, one was able to efficiently identify sera from all four DENV serotypes and to discriminate them from Zika virus positive sera.

## 1. Introduction

Dengue is currently considered as one of the most important arbovirus’ infections in the world. Almost half of the world’s population-or 3.9 billion people in 128 countries-live in areas where there is a considerable risk of Dengue virus (DENV) transmission [1,2]. Estimates show that 390 million people are infected annually, of which about 96 million result in infections ranging from minimally symptomatic to severe [3].

Dengue viruses are transmitted through the bite of the female mosquito of the species *Aedes aegypti* or other species within the *Aedes* genus [4]. The viruses belong to the Flaviviridae family-Flavivirus genus-and there are four DENV serotypes (DENV-1, DENV-2, DENV-3 and DENV-4), which are genetically and antigenically related [5]. Thus, an infection with a single serotype leads to long-lasting protection against the same serotype, and short-term protection against other DENV serotypes. However, a second infection with a different serotype may result in increased disease severity [6].

DENV has an enveloped viral particle of approximately 50 nanometers (nm) in diameter. Its genome consists of a single-stranded RNA of positive polarity and approximate size of 11 kilobases (Kb), which has an unique open reading frame (ORF) encoding a single polyprotein that is processed to yield three structural proteins (Capsid protein-C, Membrane protein-M and Envelope protein-E) and seven nonstructural ones (NS1, NS2a, NS2b, NS3, NS4a, NS4b and NS5) [7,8]. Proteins C, M and E are structural components of the virus particle that encompass and protect the viral RNA. Nonstructural proteins (NS) are related to viral multiplication, protein expression and host interaction [9].

The envelope glycoprotein (E), of approximately 50 kDa, is the largest surface component of most flaviviruses’ viral particles. It mediates viral-cellular membrane adsorption and fusion [10], and is also the primary target for neutralizing antibodies, thus, inducing protective immune responses [11].

The definitive diagnosis of DENV infections following presumptive clinical examination depends on viral isolation, detection of viral antigens or RNA in serum or tissues, or detection of specific antibodies in patient’s serum [12]. Serological methods are the most frequently used tests in the clinical practice in order to confirm a recent DENV infection [13]. Acute phase (IgM) and convalescence (IgG) antibody screenings as well as antigen screenings can be performed, for example, through the Enzyme-linked immunosorbent assay (ELISA), which stands out due to its sensitivity, simplicity and excellent cost-benefit. The intensity of the immunoglobulin response varies between the primary and secondary virus infections. Typically, during a primary infection IgM titers are much higher and more specific than during secondary infections. However, IgG titers are higher in a second infection [14]. The serological methods for immunoglobulin detection have a determinant limitation: they depend on the end of the immunological window period. This is because an infected individual may take 6 to 14 days after the onset of symptoms of the disease to generate a measurable humoral response. In addition, these antibodies have decreased sensitivity when it comes to identify genetically and antigenically related viruses, as intense cross reactions may occur among them. Thus, serology-based tests are usually unable to differentiate DENV serotypes or even distinguish DENV infections from those caused by closely related flaviviruses [15]. In this last aspect, the problems are even greater due to the emergence and concomitant circulation of Zika virus (ZIKV) in areas where DENV is already present. ZIKV presents broad antigenic similarities to DENV, making the differential diagnosis of these arboviruses difficult. The presence of Yellow fever virus (YFV) antibodies in countries where YFV vaccination is in place may also pose a diagnostic problem to Dengue diagnosis. Therefore, the goal of this work was to identify conserved and polymorphic linear B-cell DENV epitopes that could be used for diagnostic purposes without generating an increase in routine methodological complexity or operational costs for diagnosis.

## 2. Materials and Methods

### 2.1. Serum Panels

#### 2.1.1. Monoinfected Rabbit Sera

Monoinfected rabbit sera reactive to each serotype of dengue virus (DENV1, DENV2, DENV3 and DENV4), as well as uninfected sera controls, were kindly provided by Dr. Erna G. Kroon from Laboratório de Vírus, ICB/UFMG. To this end, healthy New Zealand male rabbits, at least two months old, were purchased from the Escola de Veterinária of UFMG’s Experimental Farm. For immunization, two milliliters of DENV-infected cells (viral titer between 10^5^ and 10^6^ PFU/mL) were inactivated under ultraviolet light (UV) for 5 min and then diluted in Freund’s Complete Adjuvant (Sigma, St. Louis, MO, USA) at a ratio of 4:4 mL of inoculum. This dilution, after emulsion, was inoculated into each rabbit, at four inoculation points (1 mL/point) subcutaneously. This process was repeated at least twice, at intervals of 22 to 30 days. Boosts were prepared with incomplete Freund’s adjuvant (Sigma, St. Louis, MO, USA). Blood samples were collected between 14 and 21 days after each immunization. The collected blood was centrifuged at 3000 rpm (Eppendorf 5415D Centrifuge, Hamburg, Germany) and frozen at −20 °C until testing.

#### 2.1.2. Monoinfected Mouse Sera

The co-circulation of *Dengue virus* and *Zika virus* in Brazil poses a major challenge for the correct diagnosis of infection with these viruses. Therefore, we include ZIKV positive sera in our study to assess the ability of the tests to distinguish these diseases. As a guarantee of exclusivity for ZIKV seropositivity, 7-week-old BALB/c mice were intravenously immunized with 1 × 10^6^, 1 × 10^7^ or 1 × 10^8^ PFU of ZIKV (200 µL inoculum). The virus, from the Asiatic strain, was gently provided by Profa. Dr. Silvia Sardi, from the Universidade Federal da Bahia. The virus was propagated in C6/36 strain cells (3 passages) and the viral titer obtained by plaque assay on BHK-21 cells. Sera were collected after 35 days of immunization, aliquoted and stored at −20 °C until use (UFMG’s Animal ethical review board-CEUA No. 147/2013).

#### 2.1.3. Human Sera 

DENV-infected sera samples are originated from Southeastern Brazil outbreaks, from 2006 to 2012, a period that precedes ZIKV introduction in this geographic area. Sera samples from human patients were initially tested by a DENV TaqMan^®^ RT-qPCR, as previously [16]. After patient clinical follow-up, samples were collected and serologically tested with the use of a commercial ELISA IgG kit (Panbio Dengue IgG Indirect ELISA, Alere, Waltham, MA, USA). ZIKV seropositive human samples were obtained from Fundação Ezequiel Dias (FUNED/MG). Those include sera from ZIKV seropositive women that were infected during their pregnancy and/or from their children. Samples were previously tested by molecular (Trioplex Real-time RT-PCR, CDC, Atlanta, USA) and serological (Anti-Zika Virus ELISA IgM/IgG assay, Euroimmun, Luebeck, Germany) assays.

Plaque reduction neutralization assays (PRNT50) were employed as a confirmatory post-test [17,18]. Briefly, the neutralization rate in this test is given by the ability of immune serum samples to neutralize DENV or ZIKV multiplication in VERO cells when compared to negative control serum. Sera were serially 2-fold diluted, beginning with a 1/10 up to 1/160, and added to culture media solutions containing DENV reference strains. Each dilution was tested in duplicate and the number of plaque-forming units (PFU) was recorded as the average of the number observed in each test. The PRNT50 titer is the highest serum dilution able to neutralize at least 50% of plaque formation when compared to infected cells in the absence of virus-positive serum. After PRNT validation, samples were segregated in DENV-1, DENV-2, DENV-3, DENV-4 and ZIKV positives. Samples from co-infected patients were excluded from the sera bank. The use of human sera was approved by Ethical Committees from the Faculdade de Medicina de São José do Rio Preto, SP, (FAMERP), Fundação Ezequiel Dias (FUNED) and Universidade Federal de Minas Gerais (UFMG). All procedures followed ethical guidelines in accordance to national regulations. Informed consents were obtained whenever possible.

### 2.2. Proteome Data Acquisition, Linear B-Cell Epitope Mapping and Selection of Conserved and Polymorphic Peptides 

**Figure 1:**
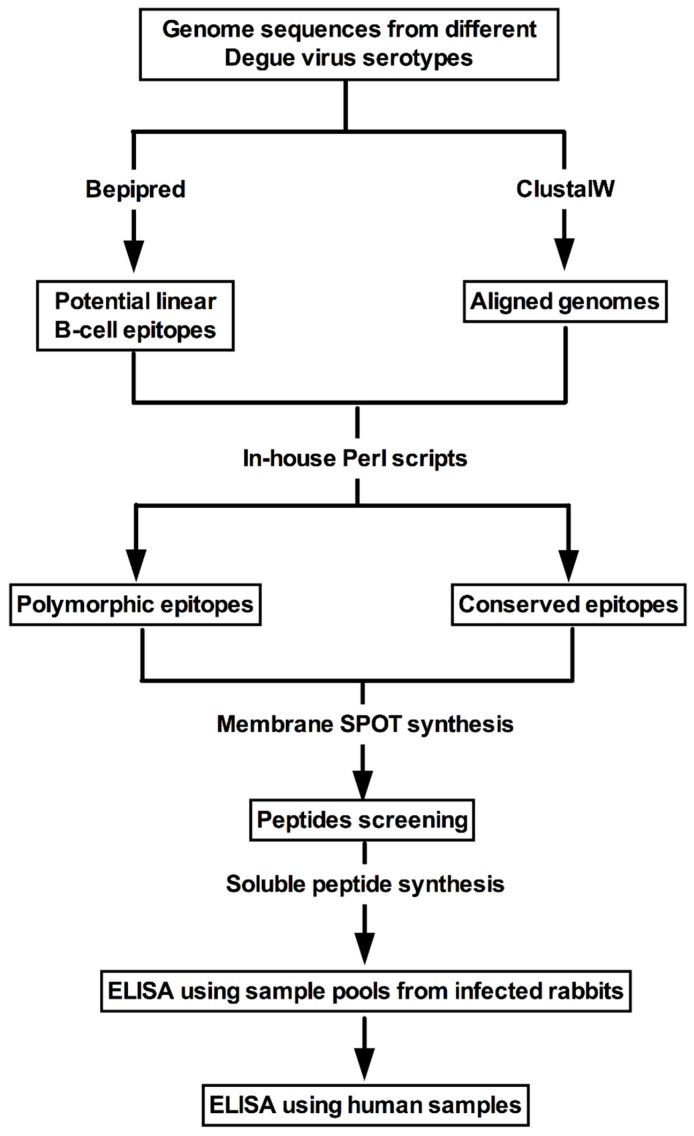
Peptide identification and selection methodology scheme.

Initially, predicted amino acid sequences from all DENV serotypes were retrieved from UniProt (release 2012_4) [19], as summarized on Table 1. B-cell linear epitopes were predicted using Bepipred (release 1.0) [20]. We used a conservative approach considering sequences at least 8 amino acids long and prediction scores above 1.3. After selection of epitopes, all polypeptide sequences were globally aligned using ClustalW 2.0 [21]. Additionally, an in-house Perl script was designed to identify conserved and polymorphic peptides among DENV proteomes from different serotypes. Peptide sequences were compared to protein sequences from ZKV, deposited in the RefSeq dataset from NCBI [22] using BLAST 2.2.1 [23]. Figure 1 represents the workflow for the identification of peptides and the ELISA test development, from selection of viral nucleic acid sequences to testing using the serum panels assembled for this study.

### 2.3. Synthesis, Spot Peptide Array and Immunoblot

Peptides were covalently synthesized in pre-activated cellulose membranes according to the SPOT synthesis technique [37]. Membranes were blocked with 5% BSA and 4% sucrose in PBS and were incubated for one hour and 30 min with diluted rabbit sera (1:500) in blocking solution. After washing, membranes were incubated with secondary anti-IgG antibodies (Sigma, St. Louis, MO, USA) diluted to 1:2000 in blocking solution and, after a second washing, developed with ECL Plus Western blotting reagent (GE Healthcare, Chicago, IL, USA). Spots were visualized by fluorescence scanning. Membranes were submitted to the same experimental conditions using sera from uninfected rabbit. Peptides with best performance were commercially, bulk-synthetized (Genone, Rio de Janeiro, RJ, Brazil). 

### 2.4. Peptide ELISA Validation with Human Samples

The Peptide ELISA (PepELISA) protocol was developed in house. We used flexible vinyl 96-well plates that were coated with 2 ug/well of water-diluted peptides. Peptides were loaded onto wells and allowed to dry overnight at 37 °C. Upon use, wells were blocked with a 5% bovine serum albumin (BSA) buffer. Sera samples were diluted in a 1:101 ratio in blocking buffer and incubated for 2 h at 37 °C. After that, anti-human IgG and IgM antibodies conjugated to peroxidase (Sigma, St. Louis, MO, USA) were diluted in 1:10,000 and 1:1000 ratios, respectively, added to the wells and incubated for 1 h at 37 °C. Before incubation with both primary and secondary antibodies, plates were washed 4 times with 0.05% PBS-Tween buffer. Plates were developed with TMB (3,3’,5,5’ tetramethylbenzidine, Sigma, USA) and reactions were stopped with H_2_SO_4_ 0.5M solution. Cutoff values were calculated by the average of true negative sera plus two-times standard deviation values (2SD) and confirmed by Receiver Operating-Characteristics (ROC) curve analysis. 

### 2.5. Statistical Analysis

Statistical analyses were performed in GraphPad Prism, version 5. In order to evaluate the potential of the selected peptides for DENV diagnostics we calculated the sensitivity, specificity, accuracy, area under ROC curve and the positive and negative predictive values [20]. The Kolmogorov-Smirnov method was used as normality test to evaluate data distribution. ANOVA tests with Bonferroni correction as multiple hypothesis tests were used to compare groups with parametric distribution and Kruskal-Wallis tests with Dunn’s corrections were used to compare data with non-parametric distribution.

## 3. Results

### 3.1. Prediction and Selection of Peptides

**Figure A1:**
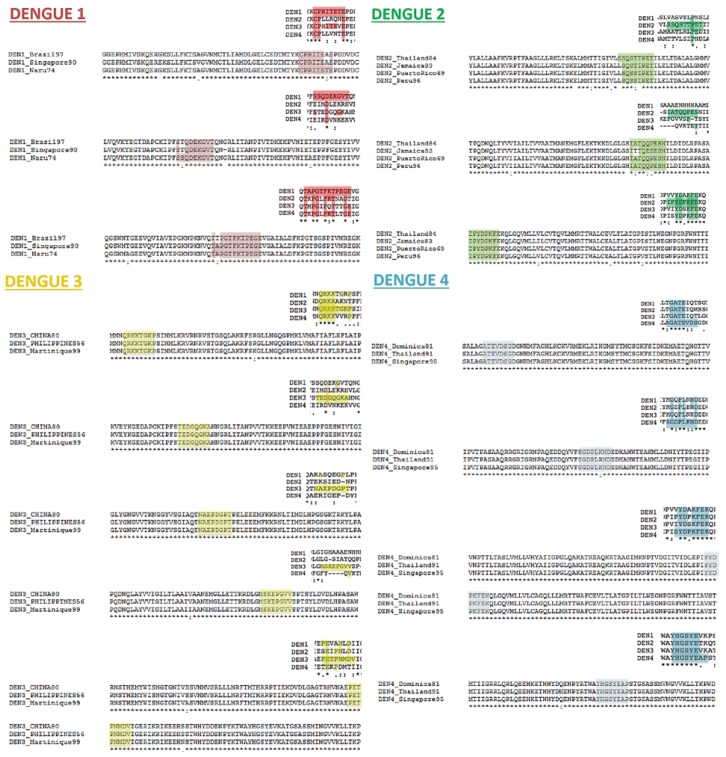
BLAST alignment with different DENV sequences utilized for the Peptide selection.

Prediction of conserved B-cell linear epitopes amongst the four DENV serotypes with specificity set to 96% or greater resulted in 9 selected sequences of 8 or more amino acids. In the case of the serotype-specific epitopes, three were selected from DENV1, three from DENV2, five from DENV3 and four from DENV4 (Table 2). Figure A1 shows the alignment of the polymorphic peptides identified in the genome of different strains of each serotype, showing their conservation between strains and the polymorphism between serotypes (in detail, upper right corner). As for the conserved epitopes, the alignment was as expected, showing great similarity between the sequences of the different DENV serotypes (not shown). Importantly, during the selection of the epitopes of interest, all sequences similar to other circulating flaviviruses, such as YFV and ZIKV, were excluded. Nine exclusively conserved DENV epitopes were selected (Table 2).

**Figure 2:**
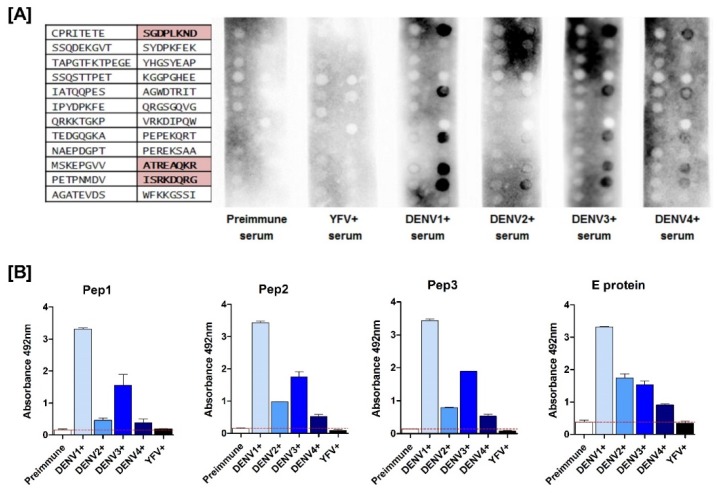
Screening of reactive peptides. (**A**) Spot blotting of nitrocellulose membranes in which peptides were synthesized. The chart on the left represents the peptide sequence for each spot. Preimmune and YFV sera did not react with any selected peptide, while DENV sera were positive for some peptides. The sequences highlighted in red are reactive for both DENV sera (1, 2, 3 and 4) and were chosen to be used in the following experiments; (**B**) Indirect IgG ELISA for validation of soluble peptides. Serum samples from infected rabbits were tested on plates with Pep1, Pep2, Pep3 and DENV3E protein (positive control). Both peptides absorbances of anti-dengue sera were higher than the preimmune control. Compared to peptides, protein E is also positive for YFV serum. Red line: Blank control.

The 24 predicted epitopes were synthesized in nitrocellulose membrane through the Fmoc strategy to evaluate its diagnostic performance. Peptides were synthesized in two 12 spot columns in duplicated membranes, as shown in Figure 2A. For peptide evaluation, sera from rabbits monoinfected with each DENV serotype were used. In order to look for cross-reaction with YFV, serum from an YFV vaccinated volunteer was included in the test. Sera from preimmune rabbits were used as internal negative controls. Tests were blindly conducted, stripping and washing the membranes before and after each tested serum.

**Figure 3:**
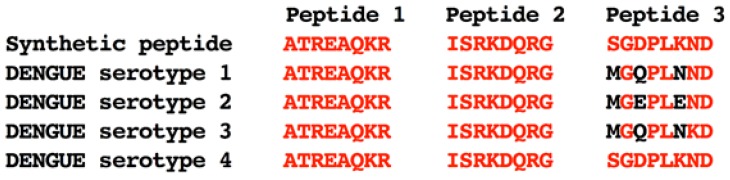
BLAST analysis of selected peptides sequences and different DENV serotypes sequences to evaluate genetic similarity between the serotypes. Only Pep3, selected as a DENV4 peptide, has three non-conserved amino acids. Conserved residues are shown in red whereas non-conserved ones are presented in black.

As expected, the preimmune and YFV positive (+) sera showed no reactivity with the spotted peptides. For DENV positive (+) sera, none of the polymorphic peptide reacted positively (Figure 2A-first row of each membrane). Two conserved peptides-sequences ATREAQKR and ISRKDQRG-were recognized by all serotype-specific sera (Figure 2A, second row of each membrane). Surprisingly, a putative DENV4 polymorphic peptide (sequence SGDPLKND) was also reactive to all DENV serotypes (Figure 2A, second row of each membrane). The three epitopes were selected for further testing (Figure 3). These were all located within non-structural regions of the viral polyprotein. The ATREAQKR peptide is derived from the NS4B protein, ISRKDQRG peptide is found within the NS5 protein; and the peptide SGDPLKND is derived from the NS3 protein.

The three peptides, now renamed Pep1, Pep2 and Pep3 (Figure 3), were commercially synthesized, purified and diluted in ultra-pure water. All three peptides were further analyzed through an indirect ELISA employing the same sera used in the spotted membrane screening. As a positive ELISA control, we used the whole viral envelope protein (recombinant DENV3E protein produced in *E. coli*); preimmune sera from the rabbits were used as negative internal controls. As observed in Figure 2B, all three peptides were recognized by DENV+ rabbit sera whereas YFV+ serum and preimmune sera did not react with the peptides.

### 3.2. Peptide Validation with Human Samples

**Figure 4:**
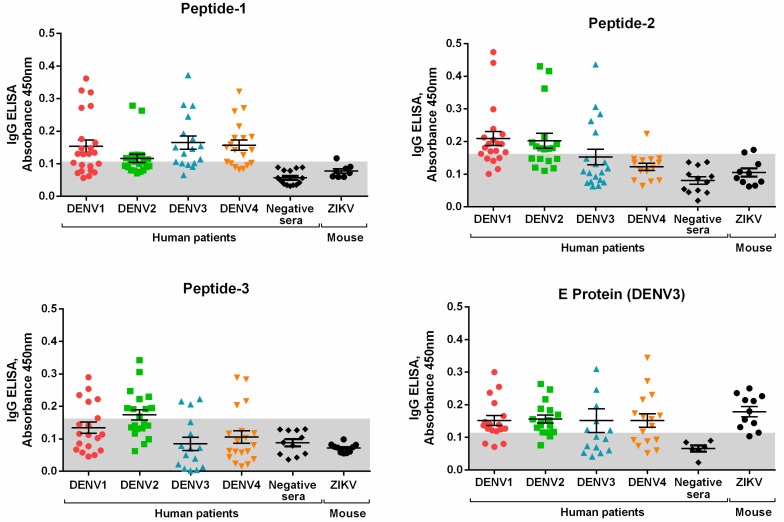
Indirect IgG Peptide-ELISA with human (DENV and negatives sera) and mouse (ZIKV sera) samples for validation. Pep1 and DENV3E protein were able to identify positive sera for all DENV serotypes, Pep2 for DENV1 and 2, and Pep3 for DENV2 only. However, DENV3E protein is the only antigen that cross react with ZIKV positive sera. Gray area: cut-off calculated for each antigen.

**Figure A2:**
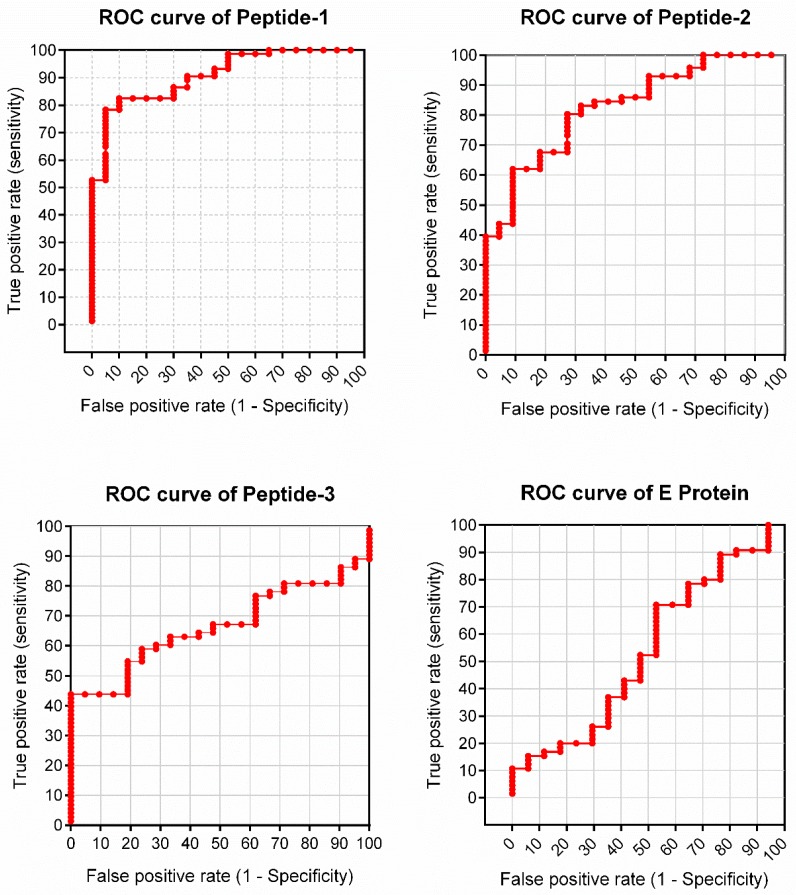
ROC curves related to ELISA presented at Figure 4.

After initial testing with rabbit sera, peptides were further evaluated using human sera and the described pepELISA. To this end, 20 DENV-positive human sera for each serotype and 20 negative sera were tested for each peptide and for the whole protein E (Figure 4). Sera from ZIKV-infected mice were also tested to compare reactivity to the selected peptides and to the whole E protein. The cutoff of the developed pepELISA was obtained by the ROC curve, which is an efficient way to demonstrate the antagonistic ratio between the signal, given by true positives (sensitivity), and the test noise, given by the false positives. The cutoff point was determined by the highest far left point of the ROC curve, which gives the highest sensitivity and specificity values (Figure A2). From the ROC curve it is also possible to analyze the Area under the Curve (AuC), a measure that represents the accuracy or overall performance of the test, as it takes into account all sensitivity and specificity values for each cut-off point. The greater the power of the test to discriminate between seropositive and seronegative individuals, the closer to the upper left corner the curve is. Conversely, the better the test, the more the area under the ROC curve approaches 1. The cutoff and AuC values are summarized in Table 3. The table also shows other statistical parameters, including the predictive negative value (PNV), positive predictive value (PPV), accuracy (AC), confidence interval (CI), number of true positives (TP), number of true negatives (TN), number of false positives (FP), and number of false negatives (FN). According to our assessment, the recombinant DENV3E protein is recognized by antisera against DENV and ZIKV (Figure 4). Therefore, the specificity of this protein in a Dengue-specific test is quite poor. Pep1 pepELISA, however, was able to identify DENV-positive sera with almost no cross-reaction to ZIKV-positive sera (Figure 4). Accordingly, this peptide presents the higher PNV, AC and AuC values. Pep2 and Pep3, on the other hand, performed poorly when compared to Pep1 and even to the whole E Protein (Table 3 and Figure 4). 

**Figure 5:**
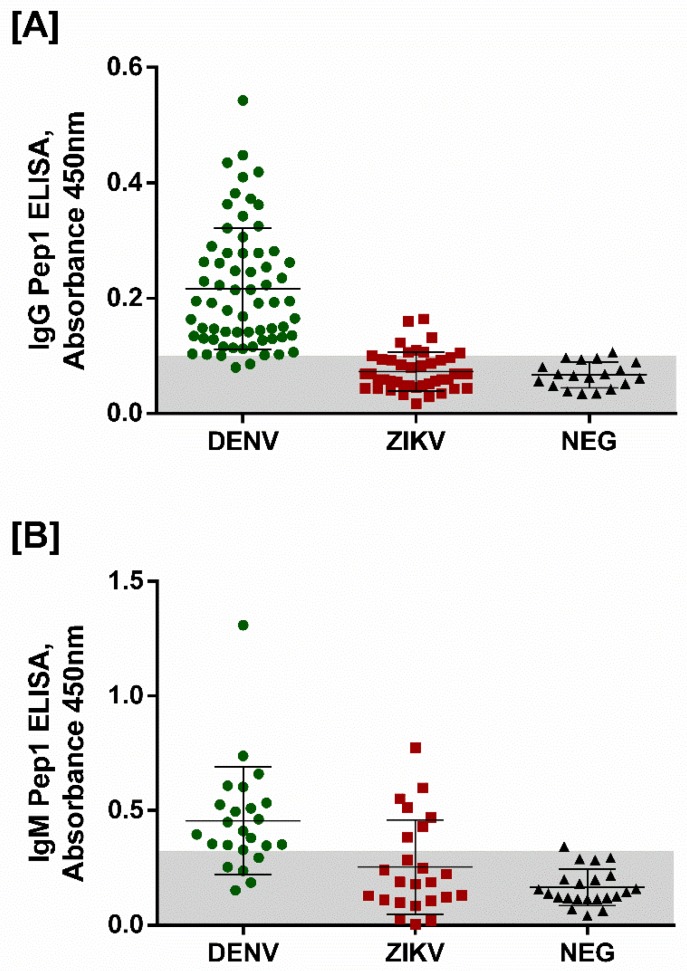
Peptide-ELISA of Peptide-1 with an open sera bank of DENV patients, ZIKV patients, and non-reactive patients–previously tested with commercial kits. (**A**) IgG Peptide 1 ELISA test, using 66 DENV positive sera, 43 ZIKV positive sera and 20 negative sera; (**B**) IgM Peptide 1 ELISA test, using 24 DENV positive sera, 24 ZIKV positive sera and 22 negative sera.

**Figure A3:**
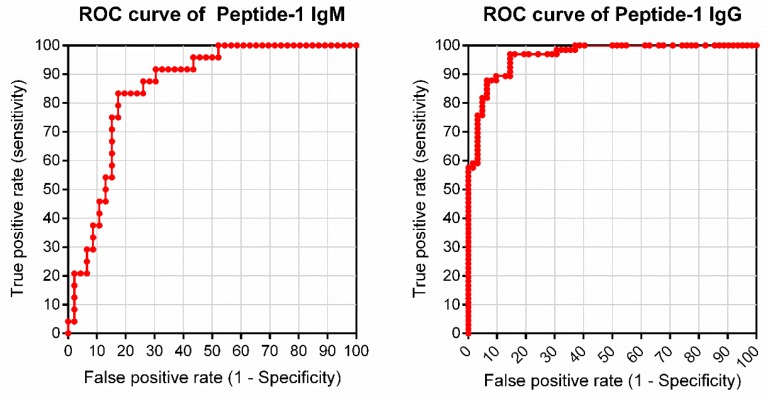
ROC curves related to ELISA presented at Figure 5.

We further evaluated the Pep1-based pepELISA using a wider human cohort that includes non-serotyped DENV seropositive patients, ZKV seropositive patients, and sera from flavivirus seronegative subjects. Figure 5A shows the anti-IgG pepELISA data plot using Pep1. As expected, the synthetic peptide was able to efficiently separate DENV + patients from ZIKV + sera. For this Pep1-based test we observed 88% sensitivity and 94% specificity. A total of 128 sera samples were used in the experiment. Figure 5B shows the results of anti-IgM pepELISA using the same peptide. In this case, the Pep1 IgM pepELISA presented 79% sensitivity and 83% specificity. Seventy sera samples were included in the testing. The values of the sensitivity, specificity, accuracy, area under the ROC curve, positive and negative predictive values, confidence interval for area under the ROC curve, true positives and negatives, false positives and negatives, are presented in Table 4. ROC curves for the IgG and IgM pep1-based pepELISAS are shown as Appendix Figure A3.

## 4. Discussion

Different arboviruses are distributed all over the world and frequently co-circulate in the same geographic region. Moreover, clinical symptoms and signs caused by dengue and other arbovirus’ infections, such as zika and yellow fever, are frequently indistinguishable among them. Therefore, differential diagnosis using laboratory techniques are necessary for public health decision making and patient management. Nonetheless, the differential serological diagnosis amongst the four serotypes of DENV, ZIKV and other arboviruses is difficult due to the existence of cross reactions between antibodies generated against one of viruses and other related pathogens, especially those caused by viruses on the same viral family, as it is the case of the flaviviruses. Serological cross-reactions are caused by structural homologies among viruses and high identity in the amino acid sequences of their major antigenic proteins [38]. Thus, the search for non-conserved regions among flaviviruses focuses on the development of diagnostic tests with greater specificity to identify the causative agent of the disease, contributing to rapid disease management and better prognostics for the patient. To this end, we have searched for non-conserved peptide regions on the DENV proteome in relation to other arboviruses—particularly related flaviviruses. 

The peptide array strategy employing SPOT-synthesis has been used as a key method for the selection of true antigenic peptides in numerous candidate epitopes from many proteins [37]. It has been used even for differential screenings between pathogens that show strong cross-reactivity in serological tests [39]. In our analysis, we identified potentially discriminatory epitopes present at the non-structural regions of the DENV polyprotein in NS4B, NS3 and NS5 regions, respectively. At first glance, the serological identification of peptides located on non-secreted non-structural proteins (unlike NS1) can be intriguing. However, several studies involving epitopes of different non-structural DENV proteins either for diagnostic or vaccine purposes have been described. As an example, Narayan and co-workers [40] described an ELISA using the DENV2-derived NS5 protein as an antigen for serotype-specific serological detection. In recent studies aimed at proving which epitopes of DENV and ZIKV proteomes were responsible for the most effective responses in B and TCD8 + activation, researchers have reported NS3, NS4, and NS5 sequences as important response generators and, thus, possible vaccine or diagnostic targets—for both DENV and ZIKV [41].

One particularity of our developed pepELISA is that optical densities (OD) are quite low for the seropositive subjects. This is not true only for the peptides but also for the whole E protein when it was used as an internal positive control of the test. Peptide based ELISAs described for other purposes have also performed similarly and that seems to be a particularity of many different peptides when they are used as antigens in immunoenzymatic assays [42,43,44]. The fact that the protein DENV3E also performed this way may indicate, however, that other technical aspects may play a role in such low OD readings, including the type of plate, blocking solutions and so on. Nonetheless, fold changes on OD readings may be taken as good parameters to evaluate the true potential of a peptide-based diagnostic system [45]. In our tests, the ratios between DENV+ and DENV—samples ranged from 3 to 7 times, whereas fold change between DENV+ and ZKV +/DENV—samples ranged from 2 to 7, considering both IgG or IgM detection (see Figure 5). Such ratios have been observed for other described peptide-based ELISAs [45].

## 5. Conclusions

In conclusion, we have identified a DENV-derived peptide with the potential to serologically discriminate dengue-positive sera from other flaviviruses using an in-house ELISA test. The peptide-based ELISA was evaluated using sera from patients living in dengue endemic regions (128 samples tested for IgG and 70 samples for IgM), and its performance was compared to the use of a DENV3E whole protein as ELISA antigen. The sensitivity and specificity values obtained using one of the tested peptides—Pep1—was comparable to values observed in studies that evaluated commercially available ELISA kits, even when using fold changes on OD readings as mains parameter for specificity. The widely used Dengue IgG detection kit Panbio^®^ Dengue IgG Indirect ELISA, for example, showed sensitivity of only 62% in endemic area [46]. Moreover, the pepELISA presented here was efficient in discriminating DENV+ from ZIK + sera, whereas the DENV3E whole protein-based ELISA was not. Indeed, recent studies show that many commercially available DENV ELISA kits present high rates of cross-reactivity to sera from acutely-infected ZIKV patients [46]. Therefore, Pep1 and the described dengue pepELISA may represent important diagnostic tools to effectively differentiate DENV from ZIKV infections in endemic regions. Nonetheless, validation and prototyping studies, as well as performances analyses compared to available Dengue and Zika ELISA kits, are still necessary to further develop the Pep1-based ELISA presented here.

## Figures and Tables

**Table 1:** Dengue virus polypeptide dataset used in this study.

Virus Serotype	Isolate Origin	Strain	Access Number	Reference
DENV1	Brazil	97-11/1997	P27909	[24]
DENV1	Singapore	S275/1990	P33478	[25]
DENV1	Nauru	West Pac/1974	P17763	[26]
DENV2	Thailand	16681/1984	P29990	[27]
DENV2	Jamaica	1409/1983	P07564	[28]
DENV2	Puerto Rico	PR159-S1/1969	P12823	[29]
DENV2	Peru	IQT2913/1996	Q9WDA6	[30]
DENV3	China	80-2/1980	Q99D35	[31]
DENV3	Philippines	H87/1956	P27915	[32]
DENV3	Martinique	1243/1999	Q6YMS3	[33]
DENV4	Dominica	814669/1981	P09866	[34]
DENV4	Thailand	0348/1991	Q2YHF0	[35]
DENV4	Singapore	8976/1995	Q5UCB8	[36]

Sequences were obtained from UniProt (release 2012_4).

**Table 2:** Polymorphic and conserved potential epitopes identified in Dengue virus genomes.

Peptide	Dengue Virus Specificity	Bepipred Score *
CPRITETE	Serotype 1	1.45
SSQDEKGVT	Serotype 1	1.30
TAPGTFKTPEGE	Serotype 1	1.53
SSQSTTPET	Serotype 2	1.34
IATQQPES	Serotype 2	1.38
IPYDPKFE	Serotype 2	1.30
QRKKTGKP	Serotype 3	1.31
TEDGQGKA	Serotype 3	1.51
NAEPDGPT	Serotype 3	2.10
MSKEPGVV	Serotype 3	1.82
PETPNMDV	Serotype 3	1.44
AGATEVDS	Serotype 4	1.43
SGDPLKND	Serotype 4	1.30
SYDPKFEK	Serotype 4	1.78
YHGSYEAP	Serotype 4	1.52
AQKRTAAG	Conserved	1.30
KGGPGHEE	Conserved	1.64
AGWDTRIT	Conserved	1.31
QRGSGQVG	Conserved	1.30
VRKDIPQW	Conserved	1.42
PEPEKQRT	Conserved	1.79
PEREKSAA	Conserved	1.41
ATREAQKR	Conserved	1.30
ISRKDQRG	Conserved	1.36

* Peptides with amino acids number >= 8 and bepipred score >= 1.3 were considered potential epitopes.

**Table 3:** Measure of diagnostic performance for Peptide-1, Peptide-2, Peptide-3 and E protein.

Parameters	Peptides			E Protein
	Peptide-1	Peptide-2	Peptide-3	
Cutoff (2SD)	0.107	0.160	0.163	0.113
TSe (%)	59.46	45.07	31.51	70.77
TSp (%)	95.00	90.91	100.00	41.18
PPV (%)	97.78	94.12	100.00	82.14
NPV (%)	38.78	33.90	29.58	26.92
AC (%)	67.02	55.91	46.81	64.63
AUC	0.9088	0.8278	0.6641	0.5367
TP	44	32	23	46
TN	19	20	21	7
FP	1	2	0	10
FN	30	39	50	19

*TSe* total sensitivity, *TSp* total specificity, *PPV* positive predictive value, *NPV* negative predictive value, *AC* accuracy, *AuC* area under the curve, *TP* true positive, *TN* true negative, *FP* false positive, *FN* false negative.

**Table 4:** Measure of diagnostic performance of IgG and IgM ELISAs for Peptide-1.

Parameters	Peptide-1	
	IgG	IgM
Cutoff (2SD)	0.111	0.324
TSe (%)	87.88	79.17
TSp (%)	93.55	82.61
PPV (%)	93.55	70.37
NPV (%)	87.88	88.37
AC (%)	90.63	81.43
AUC	0.9648	0.8542
AUC-CI95%	0.9379 to 0.9917	0.7658 to 0.9425
TP	58	19
TN	58	38
FP	4	8
FN	8	5

*TSe* total sensitivity. *TSp* total specificity. *PPV* positive predictive value. *NPV* negative predictive value. *AC* accuracy. *AUC* area under curve. *CI* confidence interval. *TP* true positive. *TN* true negative. *FP* false positive. *FN* false negative.
